# Nuclear targeting of dystroglycan promotes the expression of androgen regulated transcription factors in prostate cancer

**DOI:** 10.1038/srep02792

**Published:** 2013-09-30

**Authors:** G. Mathew, A. Mitchell, J. M. Down, L. A. Jacobs, F. C. Hamdy, C. Eaton, D. J. Rosario, S. S. Cross, S. J. Winder

**Affiliations:** 1 Department of Biomedical Science, University of Sheffield, Firth Court, Western Bank, Sheffield, S10 2TN, UK; 2 Academic Unit of Urology, Beech Hill Road, Sheffield, S10 2RX, UK; 3 Department of Oncology, Beech Hill Road, Sheffield, S10 2RX, UK; 4 Academic Unit of Pathology, Department of Neuroscience, Faculty of Medicine, Dentistry & Health University of Sheffield, Beech Hill Road, Sheffield, S10 2RX, UK; 5 Current address: Department of Ophthalmology Columbia University New York, USA.; 6 Current address: Institute of Cancer Therapeutics, University of Bradford, Bradford, BD7 1DP, UK.; 7 Current address: Musculoskeletal Medicine Division, Garvan Institute of Medical Research, University of New South Wales, Sydney, Australia.; 8 Current address: Nuffield Department of Surgical Sciences, University of Oxford, John Radcliffe Hospital, Oxford OX3 9DU United Kingdom.

## Abstract

Dystroglycan is frequently lost in adenocarcinoma, but the mechanisms and consequences are poorly understood. We report an analysis of β-dystroglycan in prostate cancer in human tissue samples and in LNCaP cells *in vitro*. There is progressive loss of β-dystroglycan immunoreactivity from basal and lateral surfaces of prostate epithelia which correlates significantly with increasing Gleason grade. In about half of matched bone metastases there is significant dystroglycan re-expression. In tumour tissue and in LNCaP cells there is also a tyrosine phosphorylation-dependent translocation of β-dystroglycan to the nucleus. Analysis of gene expression data by microarray, reveals that nuclear targeting of β-dystroglycan in LNCaP cells alters the transcription of relatively few genes, the most unregulated being the transcription factor ETV1. These data suggest that proteolysis, tyrosine phosphorylation and translocation of dystroglycan to the nucleus resulting in altered gene transcription could be important mechanisms in the progression of prostate cancer.

Dystroglycan is a ubiquitously expressed cell adhesion molecule with crucial roles in the assembly of the basement membrane^1^,^2^, in muscle integrity as a part of the dystrophin glycoprotein complex^3^,^4^, and in basolateral cell adhesion in many epithelial tissues^5^,^6^. Loss of these functional roles for dystroglycan give rise to distinct disease phenotypes including muscular dystrophies and severe neurological phenotypes^7^. Additionally, and probably as a consequence of the role of dystroglycan in branching epithelial morphogenesis^5^, loss of dystroglycan function is also associated with adenocarcinoma^8^. Mutations in dystroglycan are not known to be associated with cancer, indeed to date only one mutation in dystroglycan itself has been described, a single amino acid substitution, that gives rise to a limb girdle muscular dystrophy phenotype^9^. Moreover, dystroglycan transcription appears largely unaltered in the majority of carcinomas. The expression of glycosyltransferase such as LARGE and β3GnT1 however, which are crucially required for the post-translational modification of dystroglycan that gives it its laminin binding function, are downregulated in adenocarcinoma^10^,^11^,^12^. Furthermore many studies have reported the absence of dystroglycan protein, or the presence of aberrant short forms of dystroglycan in cancer (reviewed in^13^). Coupled with the post-translational alterations to dystroglycan; both hypo-glycosylation of α-dystroglycan and phosphorylation and proteolysis of β-dystroglycan^13^, are the more recent findings that β-dystroglycan is present in the nucleus of both normal and tumour cell lines^14^,^15^,^16^. We have therefore investigated β-dystroglycan in clinical samples from prostate tumours, the extent of β-dystroglycan loss, pattern of proteolysis, phosphorylation on tyrosine and subcellular distribution. Furthermore we have investigated the transcriptional response in LNCaP cells to the nuclear targeting of a β-dystroglycan construct.

## Results

### Dystroglycan immunohistochemistry

**Figure 1:**
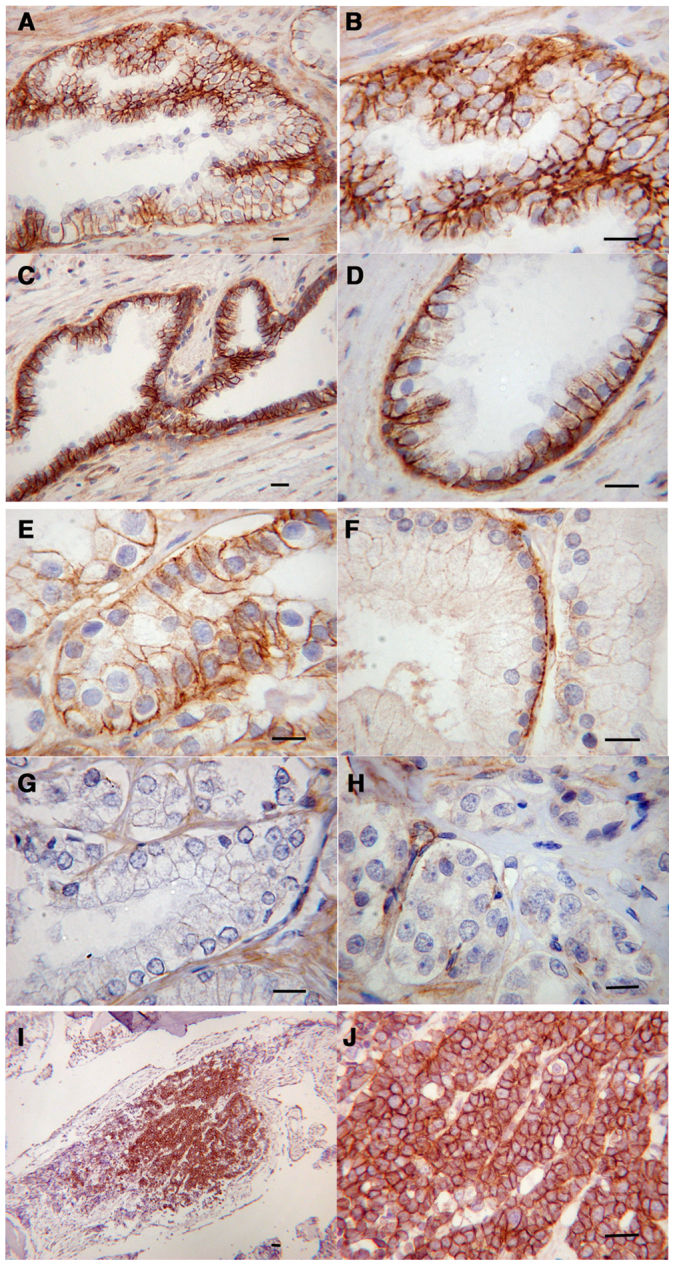
Prostate histopathology. β−dystroglycan immunohistochemistry in normal prostate (A–D), prostate cancer in situ showing varying degrees of dystroglycan loss (E–H) and in a bone metastasis sample (I), (J) where dystroglycan is re-expressed. Scale bars in all images represent 100 μm.

**Figure 2:**
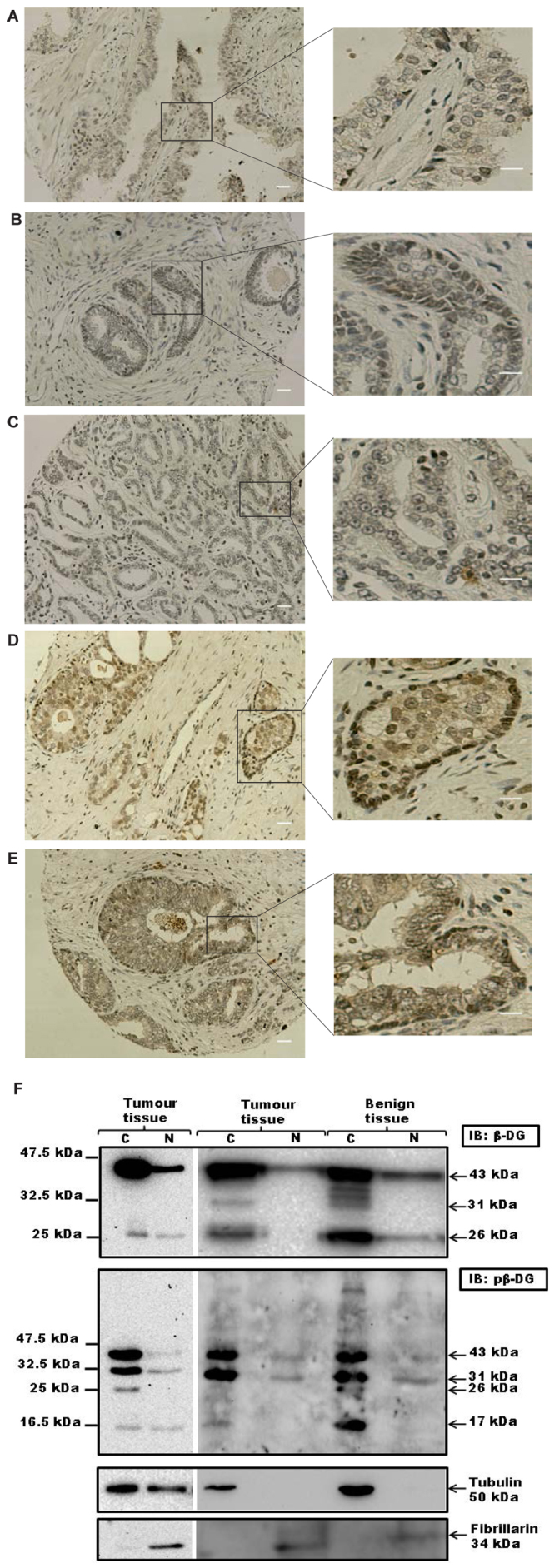
Tyrosine phosphorylated β-dystroglycan is in the nucleus of prostate epithelium *in vivo*. Immunohistochemistry of paraffin embedded TMA of prostate stained for pY890 β-dystroglycan shows a clear presence of tyrosine phosphorylated β-dystroglycan in the nucleus of prostate epithelium ((A–E) and arrows in magnified regions), scale bar is 100 um and in inset is 50 um. Cellular fractionation of prostate tissue also revealed the presence of both non-phosphorylated β-dystroglycan (F) or tyrosine phosphorylated β-dystroglycan (G) in nuclear fractions from either benign or tumour tissue. For non-phosphorylated β-dystroglycan the full length 43 kDa and cytoplasmic 26 kDa were the predominant species found in the nuclear fraction, whereas for phosphorylated β-dystroglycan the 31 kDa transmembrane and cytoplasmic fragment was more prominent in the nucleus. Fibrillarin and tubulin are shown as a nuclear (N) and cytoplasmic (C) fraction markers respectively. Both tumour samples were from hormone refractory primary TURPs.

We have investigated the expression and localisation of β-dystroglycan in tissue micro arrays (TMAs) comprising 41 cases of benign prostatic hyperplasia (BPH), 9 cases of prostatic intraepithelial neoplasia (PIN) and 113 prostate cancers. Immunohistochemistry for β-dystroglycan subunit as this tends to be less susceptible to tissue to tissue variation and the antibody is insensitive to common post-translational changes often observed in α-dystroglycan such as proteolysis or hypo-glycosylation^17^. In the epithelium in BPH and the morphologically normal background of cancer cases there was strong dystroglycan expression at the junction between the epithelial cells and the basement membrane and the intercellular junctions of epithelial cells and was indistinguishable from the staining pattern in normal tissue (Figure 1A–D). In tumour samples the intercellular expression was lost in 41% of cancers and was only weak in a further 37%. The basal expression was lost in 19% of cancers and was only weak in a further 47%. In the 9 cases of PIN there was strong staining in both sites in 3 cases, weak staining in 4 cases and intercellular absence in 2 cases. Increasing Gleason tumour grade was significantly associated with the reduction in intercellular expression of β-dystroglycan (Jonckheere-Terpstra test, p = 0.027) but not with the basal expression (p = 0.233) (Supplementary Table 1, Figure 1E–H). We also examined bone metastases samples and compared the level of dystroglycan expression in the metastasis with the level of dystroglycan in a tumour sample from the primary tissue taken from the same patients. Whilst the overall picture for dystroglycan status in the larger sample of primary tumour specimens was of a profound loss of dystroglycan immunoreactivity, the consensus from the metastasis samples was more varied. Of the 10 pairs of samples, 6 had near normal dystroglycan levels in the primary site and either showed no change in dystroglycan level between primary and metastatic site (n = 3), or showed a slight reduction from the primary to the metastatic site (n = 3). However, of the 4 samples that had reduced or absent dystroglycan in the primary tumour, the most common situation in the larger patient sample of primary only data, there was a significant upregulation of dystroglycan in the secondary metastatic site (Supplementary Table 1, Figure 1IJ). These data from patient samples follow the general scheme for dystroglycan loss and re-expression proposed for LNCaP cells undergoing EMT and MET *in vitro*^13^. One of the factors important in the proteolytic degradation and loss of dystroglycan from the cell membrane is tyrosine phosphorylation^13^,^18^. We made an analysis of 60 prostate TMA specimens using the antibody 1709 which is specific for β-dystroglycan phosphorylated on tyrosine residue 890^18^,^19^. In agreement with previous analyses of cell lines in tissue culture, a significant number of nuclei were positive for tyrosine phosphorylated β-dystroglycan (pY β-DG) (Figure 2A–E). Most often the pY β-DG immunostaining was associated with the basal cell layer where this is recognisable as such (arrows in Fig. 2). An elongated nuclear morphology similar to neuroendocrine cells of the prostate is observed in the darker staining basal cell type. In other cases however, the staining was present in luminal cells and stromal cells. Although described previously in cells maintained in vitro, this is the first report of phosphorylated dystroglycan in cellular nuclei in any intact tissue.

### Biochemical fractionation of dystroglycan

To further investigate the mechanisms and consequences of nuclear translocation of phosphorylated dystroglycan we carried out biochemical fractionation of prostate tissue samples from transurethral resection of the prostate (TURP). Western blots of extracts from both normal and tumour samples of prostate tissue revealed nuclear dystroglycan (Figure 2F,G). Detection of unphosphorylated dystroglycan using the MANDAG2 antibody revealed the majority of full length 43 kDa β-dystroglycan in the cytoplasmic fraction, with some 31 kDa (transmembrane and cytoplasmic domain) and 26 kDa (cytoplasmic domain). The 43 kDa and 26 kDa species were also detected to a limited extent in the nuclear fraction. Using an antibody specific for β-dystroglycan phosphorylated on Y890, bands of 43 kDa, 31 kDa and 26 kDa were detected in both cytoplasmic and nuclear fractions and in both normal and tumour samples. The specific finding therefore from the biochemical analysis is that the Y890 phosphorylated 31 kDa fragment of dystroglycan, comprising transmembrane and cytoplasmic domain of dystroglycan, is more frequently translocated to the nucleus.

**Figure 3:**
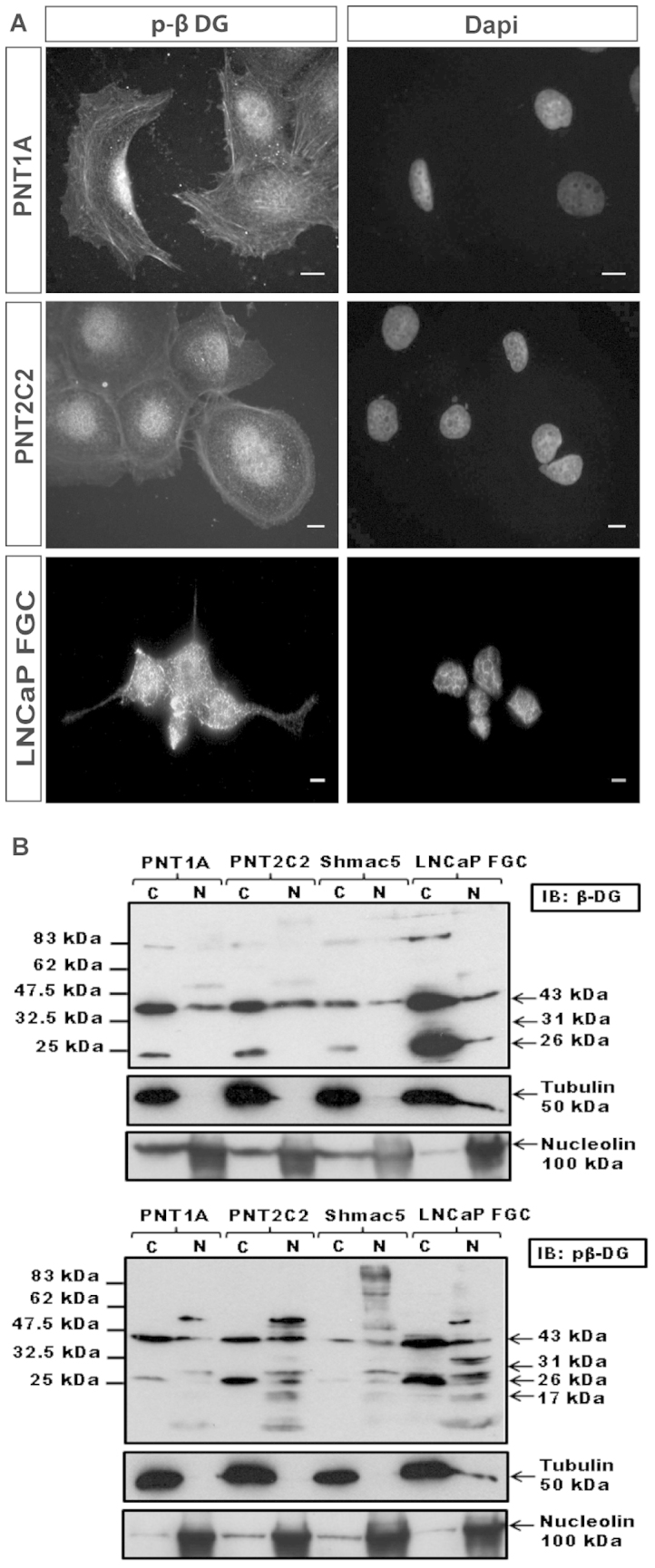
β-dystroglycan is also found in the nucleus of prostate cell lines. (A), shows immunofluorescence localisation of phosphorylated β-dystroglycan to the nucleus of LNCaP, PNT1A and PNT2C2 cells *in vitro*. DAPI staining marks the position of the nuclei. Cellular fractionation of prostate cell lines of different origins: normal epithelium, PNT1A and PNT2C2; primary tumour, Shmac5 and metastatic tumour, LNCaP, also revealed the presence of dystroglycan in the nucleus (N) fraction (B). For non-phosphorylated β-dystroglycan the predominant nuclear species was 43 kDa (upper panel) whereas the 26 kDa fragment remained in the cytoplasmic (C) fraction. Tyrosine phosphorylated β-dystroglycan (lower panel) was found more extensively in the nuclear fraction, and depending on cell line, sizes ranged from 43 kDa, 31 kDa, 26 kDa, and previously unreported 17 kDa and 50 kDa species. Tubulin and nucleolin are shown as a cytoplasmic and nuclear markers respectively.

We have demonstrated previously the cell density-dependent fragmentation profile of dystroglycan in prostate cancer cell lines including LNCaP, PC3 and DU145^13^. We therefore investigated the distribution of both phosphorylated and un-phosphorylated cytoplasmic and nuclear dystroglycan in normal and tumour cell lines from prostate. Non-phosphorylated β-dystroglycan was detected in all four cell lines tested, with the majority of the full-length protein being detected in the cytoplasmic fraction and a small amount in the nucleus, whereas the 26 kDa fragment remained cytoplasmic, and the non-phosphorylated 31 kDa fragment was not detected (Figure 3B). Analysis of the same samples for Y890 phosphorylated dystroglycan revealed the presence of full-length and 26 kDa dystroglycan in the cytoplasmic and nuclear fractions in all cell lines tested. The 31 kDa fragment was only detected in LNCaP cells however, where it was present exclusively in the nuclear fraction (Figure 3B).

### Nuclear targeting of dystroglycan

**Figure 4:**
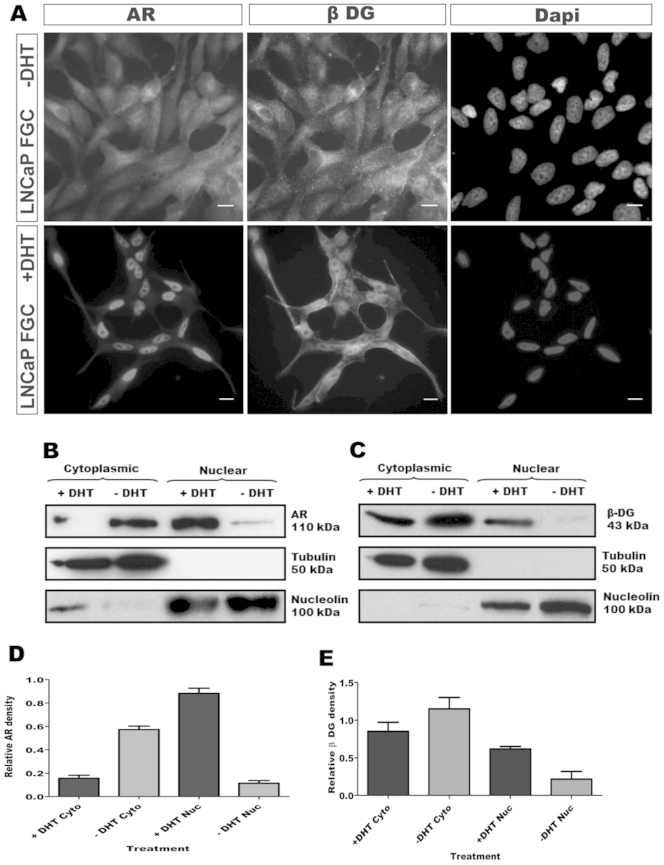
β-dystroglycan translocates to the nucleus in an androgen-dependent manner. (A), Immunofluorescence staining for androgen receptor (AR) and endogenous β-dystroglycan (β-DG) in LNCaP cells demonstrates the shift from cytoplasmic in the absence of DHT (-DHT; top panels) to nuclear in the presence of DHT (+DHT; lower panels). Both AR and β-dystroglycan are clearly cytoplasmic in the absence of DHT and nuclear in the presence, DAPI staining is shown to indicate the location of the nucleus. Fractionation of LNCaP cells into cytoplasmic and nuclear fractions (B), (C) reveals the expected dihydrotestosterone (+DHT) dependent translocation of the androgen receptor (AR) from the cytoplasmic to nuclear fraction (B). In keeping with the immunohistochemistry a significant proportion of the β-dystroglycan is also translocated to the nucleus in the presence of DHT (C). Tubulin and nucleolin are used as markers of the cytoplasmic and nuclear fractions respectively. (D), (E), quantitative analysis of three independent cellular fractionation experiments confirms the DHT-dependent shift of β-dystroglycan from cytoplasm to nucleus. N = 3 unpaired two tailed t-test with 95% confidence interval.

Several adhesion molecules undergo membrane proximal cleavage followed by translocation to the nucleus where the cytoplasmic regions exert an effect on transcription: notable examples being CD44 and notch^20^,^21^. Furthermore a previous report demonstrated androgen-dependent expression of dystroglycan and stated they had identified androgen response elements in the dystroglycan gene^22^. We therefore investigated whether androgens had any influence on the nuclear targeting of dystroglycan. We investigated LNCaP cells which have the advantage of exhibiting nuclear translocation of the 31 kDa fragment of dystroglycan (Figure 3) which recapitulates the findings from nuclear fractionation of prostate tissue (Figure 2B) and are an established androgen-responsive cell line. Dihydrotestosterone (DHT) treatment of LNCaP cells caused a robust relocation of the androgen receptor to the nucleus, as determined by both immunofluorescence microscopy and by biochemical fractionation (Figure 4ABD). Using tubulin as a cytoplasmic fraction marker, and nucleolin as a nuclear fraction marker, almost all androgen receptor (AR) was found in the nucleus following DHT treatment, with a corresponding reduction in cytoplasmic AR (Figure 4D). Unexpectedly there was a clear reproducible and significant (p = 0.019) relocation of the endogenous β-dystroglycan to the nucleus following DHT treatment (Figure 4ACE). Dystroglycan and AR were therefore targeted robustly and rapidly to the nucleus in response to DHT stimulation and appeared to co-localise in similar nuclear compartments. However, despite exhaustive efforts to immunoprecipitate dystroglycan and AR with endogenous or tagged proteins, we were unable to demonstrate any direct or indirect association between dystroglycan and the androgen receptor by these methods (Data not shown). Therefore it appears that endogenous dystroglycan can be translocated to the nucleus in an androgen-dependent manner in LNCaP cells, but not in direct association with the AR.

### Transcriptional response to nuclear targeted dystroglycan

**Figure 5:**
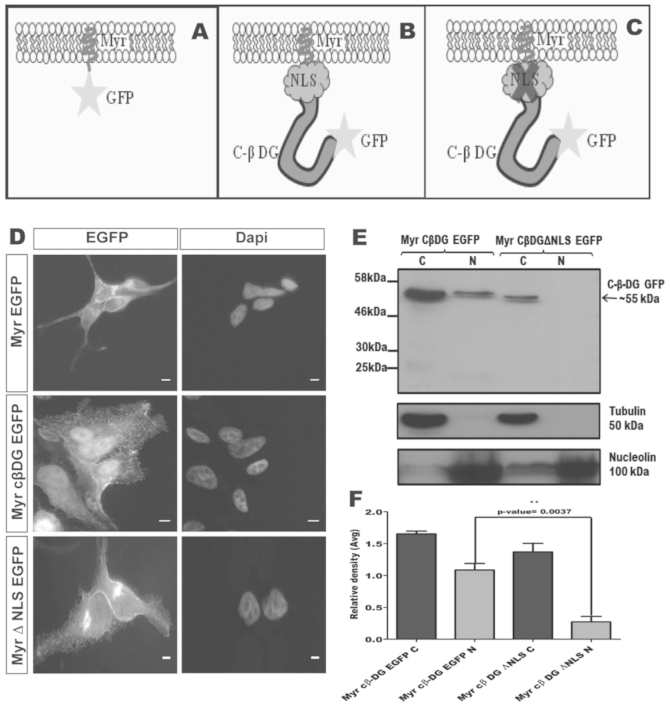
Nuclear targeting of a myristoyl-tagged cytoplasmic dystroglycan construct. Schematic diagram of myristoyl (Myr)-tagged constructs in association with the plasma membrane. (A), Myr-GFP control, (B), Myr-cytoplasmic β-dystroglycan-GFP (Myr-CβDG-GFP) with intact nulear localisation sequence (NLS), (C), Myr-cytoplasmic β-dystroglycan-GFP (Myr-ΔNLSCβDG-GFP) with mutated nulear localisation sequence. (D), Immunofluorescence localisation of the Myr-tagged constructs as detected by the GFP signal. Myr-GFP localises to the plasma membrane, Myr-CβDG-GFP localises to the membrane and nucleus, whereas Myr-ΔNLSCβDG-GFP is excluded from the nucleus, and found in cell-cell junction and a peri-nuclear compartment. Cellular fractionation and probing with GFP antibody (E) confirmed the presence of Myr-CβDG-GFP in the cytoplasmic (C) and nuclear (N) fractions, whereas the Myr-ΔNLSCβDG-GFP construct was recovered exclusively in the cytoplasmic fraction. Tubulin and nucleolin were used as cytoplasmic and nuclear markers respectively. (F) shows a quantification of 3 representative fractionation experiments, demonstrating the significantly higher levels (5-fold) of nuclear Myr-CβDG-GFP compared to Myr-ΔNLSCbDG-GFP. N = 3 unpaired two tailed t-test with 95% confidence interval.

As outlined above, CD44 and notch translocate to the nucleus in a similar manner to dystroglycan, where, in the case of CD44 and notch, they regulate transcription. With the evidence that the dystroglycan gene may contain androgen response elements^22^ and can be cleaved at the membrane^8^,^13^ and translocated to the nucleus in an androgen dependent manner (see above) we examined what the transcriptional response to dystroglycan was in LNCaP cells. In order to test more rigorously a role for dystroglycan in the nucleus we used a construct comprising an n-terminal myristoyl-tag, dystroglycan cytoplasmic domain and c-terminal GFP tag (Myr-cβDG-GFP^23^). We hypothesised that such a construct should effectively mimic the properties of 31 kDa fragment of dystroglycan in that it is membrane associated, contains the complete cytoplasmic domain sequence including NLS^24^ and it has a GFP tag to allow efficient visualisation and cell sorting (Figure 5ABC). Furthermore this construct was efficiently translocated to the nucleus without additional stimulation with androgens thus allowing any transcriptional response to dystroglycan alone to be detected without the high background of an androgen-mediated transcriptional response. In order to compare the effects of nuclear targeting on transcription, we designed a construct (Myr-cβDGΔNLS-GFP) mutated in the NLS^14^,^24^ to prevent translocation to the nucleus. Immunofluorescence analysis of LNCaP cells transiently transfected with these constructs and enriched by FACS (Supplementary Figure 1) demonstrated that whilst Myr-cβDG-GFP had both plasma membrane and nuclear localisation, Myr-cβDGΔNLS-GFP had only membrane (and Golgi) localisation, with no nuclear localisation (Figure 5D). The findings from the immunofluorescence analysis were corroborated by biochemical fractionation which revealed Myr-cβDG-GFP in both cytoplasmic and nuclear fraction of LNCaP cells, whereas Myr-cβDGΔNLS-GFP was restricted to the cytoplasmic fraction only (Figure 5EF).

**Figure 6:**
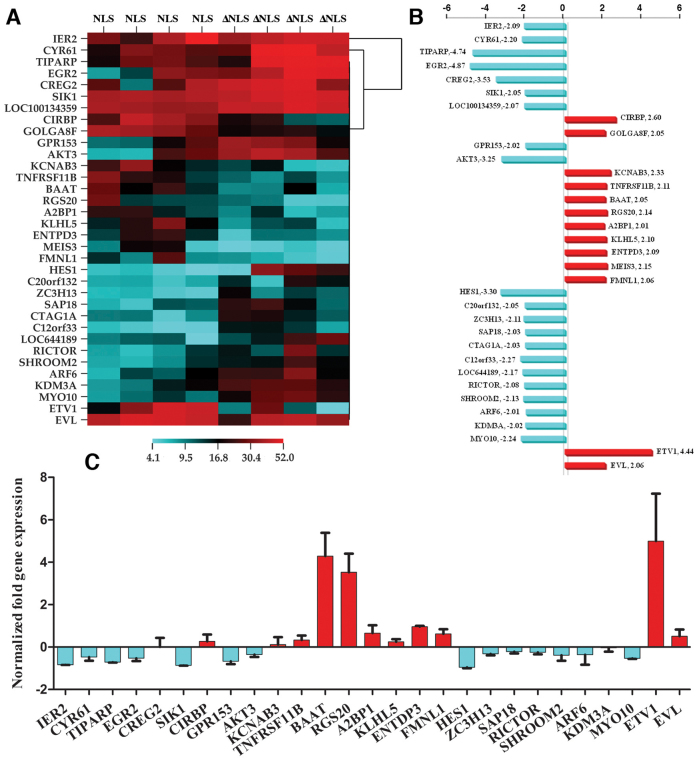
Microarray analysis of LNCaP cells with nuclear targeted or nuclear excluded CβDG. (A), Fold change in gene expression in response to dystroglycan nuclear targeting. Red bars represents an upregulation in the presence of nuclear CβDG (NLS), compared to nuclear excluded CβDG (ΔNLS), and cyan bars are a downregulation in mRNA in the presence of nuclear CβDG for all four replicates. Abbreviated gene names are indicated and the actual mean fold change in RNA shown as a bar graph in (B). (C) qPCR confirmation of mRNA levels from the original sample set used for the microarray analysis demonstrated a significant upregulation or downregulation in only 3 genes, RGS20, ETV1 and BAAT, mean ± SEM n = 4.

RNA from four independent FACS sorts of LNCaP cells stably expressing either Myr-cβDG-GFP or Myr-cβDGΔNLS-GFP (Supplementary Figure 1) was subjected to microarray analysis using the Agilent 44 K genome array. The analysis revealed a total of 13 genes upregulated by a factor of more than 2 and 21 genes downregulated by a factor of more than 2 (Figure 6). With such a small number of genes differentially expressed, pathways analysis and Ontology characterisation, performed with PANTHER^25^ with Cluster 3.0 and Treeview is not particularly informative (data not shown). In order to validate the genes that were selected by the microarray data analysis, all significant hits were subjected also to qPCR analysis from a separately stored aliquot of the original RNA samples. As can be seen from Figure 6C, qPCR analysis confirmed 3 genes in particular to be significantly altered by dystroglycan nuclear targeting; RGS20, ETV1 and BAAT. Without further in-depth analysis, it is difficult to propose a clear or intuitive function for RGS20 (regulator of G protein signalling 20)^26^,^27^ or BAAT (bile acid coenzyme A: amino acid N-acetyltransferase)^28^. ETV1 (ETS translocation variant 1) however, is an androgen responsive transcription factor which has an established role in regulation of prostate growth and prostate cancer progression^29^.

## Discussion

In the primary prostate tumour there is progressive loss of dystroglycan function through a combination of altered glycosylation of both α- and β-dystroglycan^13^ and a concomitant loss of β-dystroglycan protein through a series of proteolytic events that 1^st^ remove the extracellular domain and then the ICD^13^. Loss of dystroglycan promotes tumour growth in soft agar^13^,^22^,^30^,^31^, however for EMT, migration and invasion, some dystroglycan function is required and *in vitro* assays clearly demonstrate a requirement for dystroglycan function to be regained^13^,^22^,^31^. Furthermore, re-expression of dystroglycan to normal levels or restoration of functional glycosylation inhibits tumour properties^13^,^30^,^31^. In the present study, whilst the numbers of patient samples of metastases were limited, a significant number do show re-expression of dystroglycan in the secondary site supporting the general hypothesis above. In this regard, loss of dystroglycan during the EMT process and metastatic spread of prostate cancer, followed by re-expression of dystroglycan during mesenchymal to epithelial transition (MET) at a secondary site, mirrors findings with other cell adhesion molecules such as E-cadherin^32^.

A surprising finding was the androgen-dependent translocation of dystroglycan to the nucleus, and moreover the presence of phosphorylated dystroglycan in the nucleus of prostate epithelia *in vivo*. Although the presence of an NLS on β-DG has been well documented^24^,^33^ and the mechanisms underlying its entry into the nucleus are credible^24^, there is no comprehensive understanding of a function for the presence of dystroglycan in the nucleus thus far. It has been speculated that β-DG together with the DGC may offer stability to the nuclear membrane^16^,^34^. In silico analyses suggest that there are no predicted DNA binding regions in DG^4^, hence DG is unlikely to have a direct role in transcriptional regulation, but it may regulate transcription through association with other factors in the nucleus. The recent finding that dystroglycan has a direct role in the organisation of nuclear architecture and interaction with other nuclear proteins such as emerin and lamin B1 provides further evidence of a role for dystroglycan in scaffolding or modulation of transcriptionally active regions in the nucleus^16^. The biochemical fractionation of prostate tissue revealed the presence in the nucleus of the 26 kDa fragment of dystroglycan – equivalent to the cβDG construct used here and elsewhere^13^,^14^. Furthermore the histological analysis and the tissue fractionation also revealed the presence of tyrosine phosphorylated β-dystroglycan in the nucleus, both the full length 43 kDa form and a 31 kDa fragment equivalent to the transmembrane and cytoplasmic regions. Thus the differential translocation of the 43, 31 and 26 kDa forms of dystroglycan to the nucleus, coupled with the role of androgens, provides a potential mechanism for dystroglycan to have a regulatory role in prostate cancer progression through altering as yet unidentified nuclear functions.

The Dag1 gene itself was found to contain androgen response elements^22^, and in LNCaP cells dystroglycan expression was induced by DHT and inhibited by the anti-androgen flutamide^22^. In our own experiments where dystroglycan was targeted to or prevented from entering the nucleus, we did not see any change in dystroglycan transcript levels, but these experiments were not performed under strict androgen stimulation conditions. However, nuclear translocation of AR in response to DHT was not affected by the expression of the nuclear targeted or nuclear excluded dystroglycan constructs (Supplementary Figure 2). The LNCaP cell line is growth stimulated by treatment with DHT, so it is possible that the translocation process of dystroglycan is part of the proliferative response rather than being directly associated with mediating androgen action. Indeed, the failure to show co-immuno-precipitation between AR and dystroglycan would tend to suggest that the translocation was a result of androgen action rather than part of the androgen response mechanism. Dystroglycan is able to translocate to the nucleus in the androgen-independent prostate cell lines PC3 and DU145 (see supplementary figure 4 in reference^13^), demonstrating that dystroglycan translocation to the nucleus in prostate cancer cells is not necessarily part of the androgen response mechanism driven through the androgen receptor. However, under the conditions of our experiments, and using the androgen responsive LNCaP cell line, it is formally possible that there could be a causative link, however we have been unable to demonstrate such an association. These differences clearly warrant further investigation.

Nonetheless, microarray analysis of LNCaP cells expressing nuclear targeted or nuclear excluded dystroglycan constructs revealed a significant change in the transcription of relatively few genes. Where transcriptional changes could be validated by qPCR, ETV1 stood out as a strong candidate for a role in dystroglycan mediated modulation of prostate cancer progression. ETS factors can function as positive or negative regulators of transcription; therefore the precise balance between cancer promotion and inhibition by ETS factors may control cancer progression by differentially regulating specific target genes/oncogenes^35^. Furthermore, the fusion between androgen-regulated TMPRSS2 and ETS transcription factor gene ERG or ETV1 is the most frequent genetic alteration that occurs in 40–70% of CaP^36^. It has been demonstrated that aberrant expression of ETV1 is not sufficient to initiate neoplastic transformation but instead may cooperate with other genetic events to promote prostate cancer progression^37^.

The LNCaP cell line used in these studies carries both a mutated androgen receptor, with promiscuous steroid binding activity and a TMPRSS2-ETV-1 fusion^38^,^39^. Both these features could affect androgen responses and the dystroglycan translocation process. Studies to determine the involvement of dystroglycan nuclear translocation in response to androgen in other cell lines with differing sensitivities to androgen such as DuCap and PC3 expressing wildtype AR, are ongoing.

Advanced stages of CaP are associated with the expression of ETS1 and ETS2. The transcriptional activation of the ETS genes is necessary for the upregulation of ECM degrading proteases such as MMP-1 and MMP-9^40^. The majority of ETS gene fusions are hormone regulated, thus explaining the pathogenesis underlying exquisitely hormone sensitive CaP^41^. The recurrent fusions of the 5′ untranslated region of the TMPRSS2 genes to ERG and ETV1 is seen in majority of CaP samples and in cell lines containing the TMPRSS2/ETV1 fusion gene, androgen appears to play a role in mediating ETS overexpression^42^,^43^. Gene microarray studies were used to show that ETV1 is a novel androgen regulated gene^29^. ETV1 mRNA and protein are upregulated in response to ligand activated AR in LNCaP cells, but there is no detectable ETV1 expression in normal prostate cells. The ETV1 promoter was shown to be induced by androgens and recruits AR in the context of chromatin; this in response leads to ETV-1 regulated endogenous MMP gene activation^29^. Remarkably, the disruption of ETV1 expression in both androgen dependent and independent CaP cells significantly compromises the invasive capacity of the cells, suggesting a significant role of ETV1 in CaP metastasis^29^. Interestingly, the up regulation of ETV1 gene on nuclear translocation of cβ-DG could provide a mechanism for the androgen mediated regulation of nuclear dystroglycan function. One further aspect of dystroglycan and ETV1 function that has not been addressed is signalling through the ERK/MAP kinase pathway. Dystroglycan is known to be a scaffold for the ERK/MAP kinase pathway^44^ which can further direct growth factor and Ras mediated signalling to specific cellular compartments^45^. There is clear evidence of a role for oncogenic ETS transcription factors including ETV1^46^ in the progression of the majority of prostate cancers. Furthermore oncogenic ETV1 can mimic Ras/MAPK signalling in prostate cancer leading to increased cell migration^47^. Therefore the combined effects of dysregulation of dystroglycan function through proteolysis leading to altered cell adhesion and motility, changes in the scaffolding of MAPK signalling, nuclear translocation of dystroglycan coupled with increased ETV1 transcription, which also activates MAPK-dependent signalling that itself leads to an increase in cell migration, represents a positive feedback mechanism driving prostate cancer progression that warrants further investigation.

## Methods

### Histopathology

Triplicate samples from 163 anonymised archival cases of both normal and neoplastic paraffin wax-embedded tissues were arrayed into tissue microarrays using a 0.6 mm punch (Beecher Instruments Inc., Sun Prairie, US). Approval was obtained for this study from the University of Sheffield Medical Research Ethics Committee (MREC) approval number MREC/01/4/061. Informed consent was obtained from all donors. Antigen retrieval, primary antibody staining and detection and counterstaining were performed and analysed as described previously^8^. A further 10 samples of matched pairs of primary prostate carcinoma in situ and bone metastases were processed separately. Slides were examined by SSC, an experienced histopathologist, but blind to any case details apart from any obvious morphology and scored for Gleason tumour grade and the level of intercellular and basal β-dystroglycan staining as compared to normal control tissue. Samples of frozen human prostate tissue (benign and tumour) obtained by transurethral resection of the prostate (TURP) were provided by the Prostate Cancer: Mechanisms of Progression and Treatment collaborative (ProMPT; Sheffield). A separate tissue microarray containing 60 tumour samples of Gleason Grade 3 or above were stained for tyrosine phosphorylated β-dystroglycan using antibody 1709 under the same conditions as described previously^8^.

### Cell fractionation from tissue

Benign and prostate cancer (hormone refractory) tissue harvested from anonymised TURP specimens and stored in ice cold RPMI-1640 media was obtained from the tissue procurement facility at the Department of Urology, Royal Hallamshire Hospital, Sheffield, UK. Shortly after receiving the samples, the tissue specimens were minced finely into 3–5 mm^3^ pieces and washed with RPMI-1640 at 4°C for 10 mins. Cell fractionation was performed using a subcellular tissue fractionation kit (Thermo Scientific) according to the manufacturer's instructions.

#### Cell culture and generation of stable lines

LNCaP FGC cells^48^ were maintained as described previously^13^. For experiments requiring androgen treatment LNCaP cells were grown to about 70% confluence and then cultured for 48 hrs in phenol red free RPMI 1640 supplemented with 1% charcoal stripped FBS. Following androgen starvation, the cells were treated overnight (16–18 hrs) with 10 nM Dihydrotestosterone (DHT) in ethanol. An equivalent amount of ethanol vehicle was also added to controls. N-terminal myristoylated β-dystroglycan cytoplasmic domain constructs^23^, were transfected into LNCaP cells as described previously^13^ and subjected to FACS utilising the C-terminal GFP tag (Supplementary Figure 1). PNT2-C2 and PNT1A cells, normal immortalised human prostate epithelial cell lines^49^ were cultured in RPMI 1640 supplemented with 10% FBS. Shmac5 cells, a cell line derived from a moderately differentiated primary prostate tumour^50^ were cultured in keratinocyte serum free media supplemented with 2% FBS, 5 ng/ml epidermal growth factor and 25 μg/ml bovine pituitary extract.

#### Nuclear fractionation

Cells were rinsed in cold PBS and chilled on ice and harvested in the minimum volume of cold buffer I (0.32 M sucrose, 10 mM Tris-HCl pH 8.0, 3 mM calcium chloride, 2 mM magnesium acetate, 0.1 mM EDTA, 0.5% NP-40, 1 mM DTT, 0.5 mM PMSF and complete protease inhibitor mixture). Harvested cell lysates were homogenized using a cold dounce homogenizer on ice and spun at 600 g for 10 mins at 4°C, pellet and supernatant were retained. The pellet was resuspended in buffer I and mixed with an equal volume of buffer II (2 M sucrose, 10 mM Tris-HCl pH 8.0, 5 mM magnesium acetate, 0.1 mM EDTA, 1 mM DTT, 0.5 mM PMSF and complete protease inhibitor mixture). The resulting mixture was then carefully overlayed onto 1.8 M sucrose and nuclei were recovered by centrifugation at 30,000 g for 50 mins. The nuclear pellet was resuspended directly in SDS-PAGE sample buffer and used as the nuclear fraction in immunoblotting experiments. The retained supernatant was centrifuged at 9300 g for 10 mins at 4°C and the resultant supernatant was used as the cytoplasmic fraction. Adapted from^51^.

#### Immunofluorescence microscopy and western blotting

LNCaP cells were fixed and stained for microscopy as described previously^52^. Widefield microscopy was carried out on a Leica DMIRE2 fitted with a DC350F digital monochrome camera. Multi-channel fluorescent images were re-combined post acquisition using Adobe Photoshop. Confocal images were taken on a Leica TCS SP1 laser scanning confocal microscope. SDS-PAGE and western blotting was carried out as described previously^52^ and quantitative analysis was performed using unpaired two tailed t-test with 95% confidence interval. The following antibodies were used for blotting (WB) and/or immunofluorescence (IF) applications at the indicated dilutions. Actin (Santa Cruz; WB 1:1000), androgen receptor (N-20 Santa Cruz, WB 1:1000, IF 1:100), β-dystroglycan (MANDAG2; WB 1:100, IF 1:50), β-dystroglycan (43DAG/8D5 Novocastra, WB 1:100, IF 1:50) tyrosine phosphorylated β-dystroglycan (1709^19^, WB 1:1000, IF 1:50) nucleolin (MS-3 Santa Cruz, WB 1:3000) tubulin (Sigma, WB 1:2500), fibrillarin (Cell Signalling WB 1:1000). Species specific secondary antibodies conjugated to horseradish-peroxidase (Sigma, 1:10000) were used to detect western blots by ECL, and TRITC or FITC-conjugated (Vector, 1:100) for immunofluorescence microscopy. Cell nuclei were counterstained with DAPI.

#### Microarray and qPCR

LNCaP cells stably expressing Myr-cβDG-GFP and Myr-cβDGΔNLS-GFP were grown to 90% confluency, trypsinised and resuspended in FACS buffer. Cells were sorted using a Mo-Flo FACS (Dako Cytomation) using the GFP signal at 488 nm and fluorescence detected using a 531/40 nm bandpass filter. A total of 4 independent samples of approximately 4 × 10^6^ cells were sorted for each cell line (Supplementary Figure 1). 2 × 10^6^ cells were pelleted and immediately processed using the nuclear and cytoplasmic fractionation protocol as above and 2 × 10^6^ cells were used for total RNA isolation using the Stratagene Absolutely RNA™ kit. RNA concentration was analysed using a NanoDrop spectrophotometer. Total RNA was divided into two aliquots; one for microarray analysis and one for q-PCR, and stored at −80°C until use.

For microarray analysis: mRNA (Supplementary Figure 1) was amplified, labelled and hybridized on Agilent 44 K human genome array slides (4 × 44 K format; MoGene MO, USA). Microarray data was analysed using Agilent Feature Extraction which provides Linear and LOWESS dye normalization. The data was then read by GeneSpring which summarised duplicate probes and combined the data from the separate arrays into a single table including the annotation information from the array design. Finally, Fold Change and Regulation columns are added in Excel for estimation of gene expression and normalisation. Differentially expressed genes across the two experimental conditions were identified based on fold change equal or greater than 2.0 and p-value equal to or less than 0.05, clustered and visualised using Cluster Analysis^53^ and Heatmap Builder^54^.

For qPCR: total RNA isolated from the sorted cell populations was reverse transcribed to cDNA using SuperScript™ III first strand synthesis system. Relative gene expression of differentially expressed genes obtained from the microarray analysis was performed using gene specific primers with SYBR green real-time PCR master mix in triplicate (Supplementary Table 2). The data were analysed by iCycler iQ software by normalising to GAPDH and 2^ΔΔCt^ method was used to calculate the relative gene expression by comparing Myr-cβDG to the reference sample of Myr cβDGΔNLS.

## Author Contributions

G.M. prepared figures 2–6. J.M.D. produced data for Figure 1 with assistance from A.M. D.J.R. provided samples and data for figure 2. S.S.C. analysed data in Figure 1,2 and STable 1. L.A.J. produced SF2. S.J.W. co-directed and obtained funding for the study with C.E. and F.C.H. S.J.W. wrote the main manuscript with G.M. and C.E. All authors reviewed the final manuscript.

## Supplementary Material

Supplementary Information Supplementary
