# Supplementary material for: Nuclear targeting of dystroglycan promotes the expression of androgen regulated transcription factors in prostate cancer
Source: Sci Rep. 2013 Sep 30;3:2792. doi: 10.1038/srep02792 (PMC3786294; doi:10.1038/srep02792)
Supplement: Supplementary Information — Supplementary [file srep02792-s1.pdf]

Nuclear targeting of dystroglycan promotes the expression of androgen regulated transcription factors in prostate cancer

G. Mathew[1,2], A. Mitchell,[1,3] J.M. Down,[4] L.A. Jacobs,[1] F.C. Hamdy,[4,5] C. Eaton,[4] D.J. Rosario,[6] S.S. Cross,[7] & S.J. Winder[1]*

[1]Department of Biomedical Science, University of Sheffield, Firth Court, Western Bank, Sheffield, S10 2TN, UK.

[4]Academic Unit of Urology, [6]Department of Oncology and [7]Academic Unit of Pathology, Department of Neuroscience, Faculty of Medicine, Dentistry & Health University of Sheffield, Beech Hill Road, Sheffield, S10 2RX, UK.

Present addresses:

[2] Department of Medical and Molecular Genetics, Indiana University School of Medicine, IUPUI, Indianapolis, IN 46202, USA

[3]Institute of Cancer Therapeutics, University of Bradford, Bradford, BD7 1DP, UK

[5]Nuffield Department of Surgical Sciences, University of Oxford, John Radcliffe Hospital, Oxford OX3 9DU United Kingdom

*Author for correspondence:

s.winder@sheffield.ac.uk

tel +44 114 222 2332

Supplementary Information

A

Tissue	BPH (41)	PIN (9)	Carcinoma (113)
DG staining pattern			
Basal - normal	100	33	34
Basal - weak	0	66	47
Basal - absent	0	0	19
Intercellular - normal	100	33	22
Intercellular - weak	0	44	37
Intercellular - absent	0	22	41

B

DG staining in primary tumour	DG staining in bone met	Relative change	Number
Normal	Normal	None	3
Weak	Weak		
Normal	Weak/absent	Down	3
Weak	Normal	Up	4
Absent	Weak		
Absent	Normal		

Supplementary Table 1 Summary data for prostate histopathology. A, β-dystroglycan staining pattern in 41 cases of benign prostatic hyperplasia (BPH), 9 cases of prostate intraepithelial neoplasia (PIN) and 113 cases of carcinoma in situ. Staining was scored blind as either normal, weak or absent in basal and intercellular regions. B, β-dystroglycan

staining pattern in 10 matched pairs of primary prostate carcinoma and bone metastasis. Staining intensity is compared in metastasis relative to primary as either no change, down or up.

Supplementary Table 2 qPCR primers

qPCR primers

GENE (UP)	SEQUENCE (5′-3′)	PRIMER LENGTH	AMPLICON	Tm	LOCATION (cDNA)
**(1) ETV1**					
**FwdP**	GGCCCCAGGCAGTTTTATGAT	21	113	62.5	769-789
**RevP**	GATCCTCGCCGTTGGTATGT	20		61.7	881-862
**(2) CIRBP**					
**FwdP**	AGGGCTGAGTTTTGACACCAA	21	123	61.8	33-53
**RevP**	ACAAACCCAAATCCCCGAGAT	21		61.4	155-135
**(3) KCNAB3**					
**FwdP**	AGGGAAGTCTGGTCTTCGGG	20	113	62.8	249-268
**RevP**	CATGCTCATAGGCTACAGTCAG	22		60	361-340
**(4) MEIS3**					1-20
**FwdP**	ATGGCCCGGAGGTATGATGA	20	198	62.4	198-179
**RevP**	GAAGAGCGGGTGTCCATAGA	20		60.7	
**(5) RGS20**					
**FwdP**	CAGATGGGATCAGAGCGGATG	21	166	62.1	70-90
**RevP**	GGTTTCTAACAGTGAGACACGAG	23		60.5	235-213
**(6) TNFRSF11B**					
**FwdP**	CACAAATTGCAGTGTCTTTGGTC	23	216	60.7	489-511
**RevP**	TCTGCGTTTACTTTGGTGCCA	21		62.5	704-684
**(7) KLHL5**					
**FwdP**	GCTTCATCCATCCAACTGTCTT	22	153	60.3	765-786
**RevP**	GCAATTTCGCTGGCTGGTAATAA	23		61.7	917-895
**(8) ENTPD3**					
**FwdP**	GACTTTAGGGGTGCTCAAATCA	22	108	60.0	508-529
**RevP**	CATGTGCCACAGGTTCTTCTC	21		60.9	615-595
**(9) FMNL1**					
**FwdP**	CACCTGACCATCAAGCTGACC	21	104	62.7	601-621

**RevP**	CGTAGGCACATAATACAGACGTG	23		60.8	704-682
**(10) EVL**					
**FwdP**	CTTCCGTGATGGTCTACGATG	21	133	60.0	38-58
**RevP**	TGCAACTTGACTCCAACGACT	21		61.9	170-150
**(11) BAAT**					
**FwdP**	GGAGGGGATTATATGGGAGTCC	22	168	60.6	199-220
**RevP**	TGGAGCACTGGCAACTTTATTG	22		61.1	366-345
**(12) A2BP1**					
**FwdP**	GCACACATTAAACCTGTACCCT	22	110	60.2	180-201
**RevP**	GTGCTGCGTCATCTGTCTGT	20		62.5	289-270

**GENE (DOWN)**	**SEQUENCE (5′-3′)**	**PRIMER LENGTH**	**AMPLICON**	**Tm**	**LOCATION (cDNA)**
**(1) EGR2**					
**FwdP**	TCTTCCCAATGATCCCAGACT	21	154	60.0	506-526
**RevP**	TTACGGATTGTAGAGAGTGGAGT	23		60.0	659-637
**(2) TIPARP**					
**FwdP**	ACGAGTGGTTCCAATCCAAGA	21	103	61.1	396-416
**RevP**	TGGGTGCAAAAGATCAGTCTG	21		60.2	498-478
**(3) CREG2**					
**FwdP**	GCGCTGCTAGAGGATTCGG	19	142	62.6	181-199
**RevP**	GCCGGTAGGAGAACATCCC	19		61.5	322-304
**(4)HES1**					
**FwdP**	ACACGACACCGGATAAACCAA	21	150	61.7	65-85
**RevP**	GCCGCGAGCTATCTTTCTTCA	21		62.5	214-194
**(5)AKT3**					
**FwdP**	AATGGACAGAAGCTATCCAGGC	22	130	61.8	290-311
**RevP**	TGATGGGTTGTAGAGGCATCC	21		61.3	419-399
**(6)MYO10**					
**FwdP**	AAGTGGGGCAGGTAAAACCG	20	114	62.4	474-493
**RevP**	GCTCGTTCAACACAGGATGTC	21		61.2	587-567
**(7)CYR61**					
**FwdP**	CTCGCCTTAGTCGTCACCC	19	220	61.8	25-43

**(8) SHROOM2**					
**FwdP**	CGAACCTACAGCGCACCTTAG	21	202	62.8	488-508
**RevP**	GACGTGTCGGCTTTGGACAA	20		63.0	689-670
**(9) ZC3H13**					
**FwdP**	TCTGATAGCACATCCCGAAGA	21	90	60.1	49-69
**RevP**	CAGCCAGTTACGGCACTGT	19		62.2	138-120
**(10) IER2**					
**FwdP**	ACTGGTCCCGAGCAAGAAAG	20	68	61.8	402-421
**RevP**	CGACTTCGGATGACGCTCC	19		62.5	469-451
**(11) RICTOR**					
**FwdP**	TCCAAAGACTCGACAGTATGTGC	23		62.2	711-733
**RevP**	GGCTAGAAATCGTGCTTCTCTG	22		60.7	849-828
**(12) SIK1**					
**FwdP**	GCTTCTGAACCATCCACACAT	21	124	60.3	237-257
**RevP**	GTGCCCGTTGGAAGTCAAATA	21		60.6	360-340
**(13) SAP18**					
**FwdP**	GGGGAAATGTACCGTCCAGC	20	171	62.6	200-219
**RevP**	CCTTAACTCGATAGCCAGGTCTT	23		61.2	370-348
**(14) KDM3A**					
**FwdP**	ACACCGACGTTACCAAGAAGG	21	150	62.0	158-178
**RevP**	CAGGTGACTTTCGTTCAGCTAA	22		60.2	307-286
**(15) GPR153**					
**FwdP**	ACGACGAGGAGTCAGACGAT	20	105	62.2	959-978
**RevP**	GGGCCACAAAATCACCTCCAT	21		62.7	1063-1043
**(16) ARF6**					
**FwdP**	CCGGCAAGACAACAATCCTGT	21	122	62.9	71-91
**RevP**	CACATCCCATACGTTGAACTTGA	22		60.5	192-170

The Q-PCR reactions were set in an iCycler iQ machine and cycling conditions of 94^{0}C for 3mins (hot start) followed by 40 cycles of 95^{0}C for 15sec, 60^{0}C for 60sec and 72^{0}C for 30sec was used. In order to confirm the presence of a single PCR product an additional

dissociation step at 72^{0}C was performed. PCR reactions for each gene was set in triplicates and qPCR was performed thrice per gene. The iCycler iQ software was used to analyse the data; samples were normalised to GAPDH and 2(-ΔΔCt) method was used to calculate the relative gene expression. The relative expression level was calculated by comparing the level of Myr C-β DG to the reference sample of Myr Δ NLS DG.

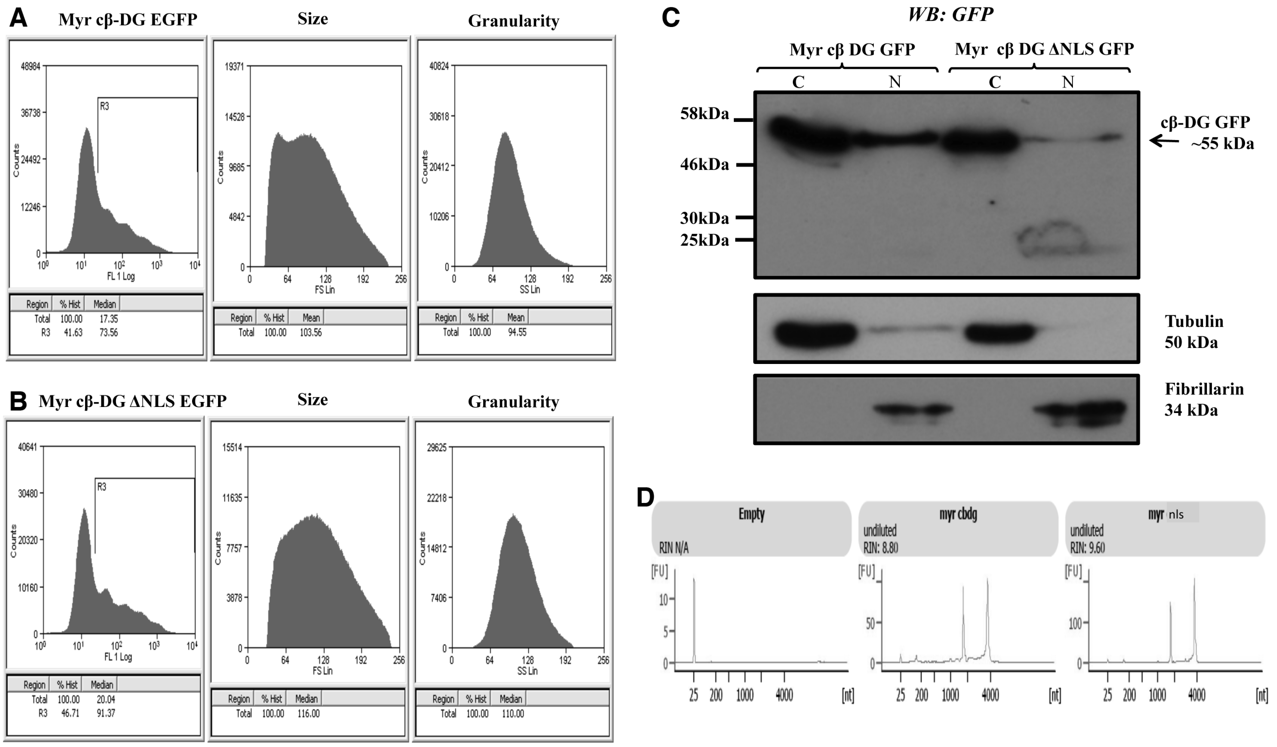

Supplementary Figure 1

A. FACS characteristics of LNCaP cells stably expressing Myr-CβDG-GFP (A) or Myr-ΔNLSCβDG-GFP (B) used for the microarray analysis. C, cell fractionation of FACS sorted cells demonstrating the virtual absence of Myr-ΔNLSCβDG-GFP, as determined by GFP immunoreactivity, from the nuclear faction of LNCaP cells. Tubulin and fibrillarin mark the non-nuclear and nuclear fractions respectively. D. RNA quality analysis for RNA samples used in the microarray hybridisation.

Cells with EGFP fluorescence were selected by setting up a gate to select EGFP positive cells. The EGFP fluorescence intensity for sorting was determined by gating the brightest cells emitting maximum signal. EGFP positive cells were further selected on the basis of the FSC (forward scatter channel) and SSC (side scatter channel) scattergram to sort cells expressing Myr C-βDG EGFP or Myr C-βDG ΔNLS EGFP constructs.

To prevent degradation of RNA, total RNA isolation was performed immediately after obtaining cells from FAC sorting and stored at -80^{0}C. Approximately 1μl of total RNA was used for determining the RNA concentration using the NanoSpec 3000 spectrophotometer.

Approximately 1 μg of total RNA from each sample at a concentration of 100 ng/ul in a volume of 10 μl was sent for microarray analysis. Further analysis of the RNA quality control and usability was implemented at the MoGene LLC facility using the Agilent 2100 bioanalyzer prior to using the samples for microarray analysis. C illustrates fluorescent analysis of RNA migration. The electropherograms (fluorescent units vs nucleotides) represent the RNA integrity value (RIN) for the experimental and control samples used for microarray analysis.

Supplementary Figure 2. The androgen receptor is still translocated to the nucleus in response to androgens in both Myr-CβDG-GFP and Myr-CβDGΔNLS-GFP expressing cells.

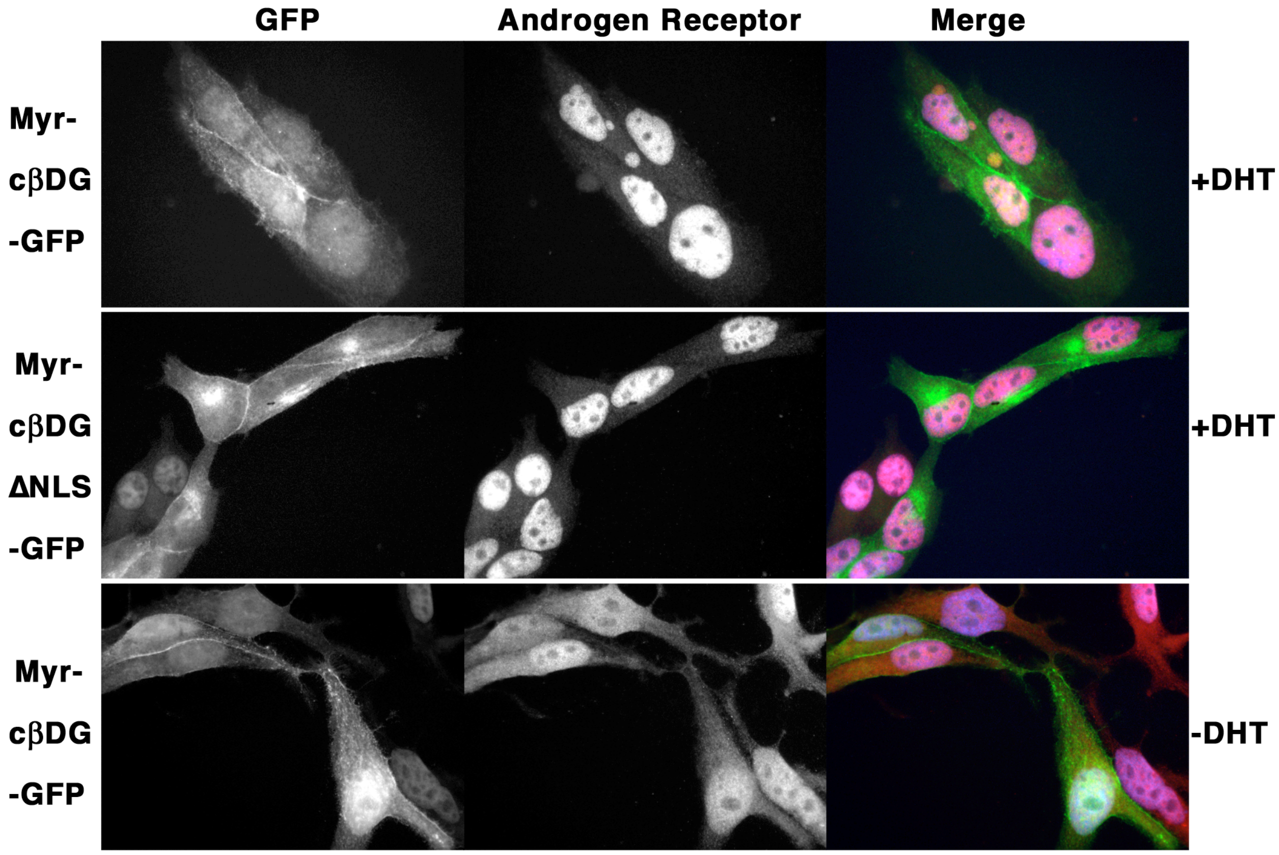

LNCaP cells stably expressing Myr-CβDG-GFP and Myr-CβDGΔNLS-GFP were serum starved for 48 hours prior to the addition of 100nM DHT (+DHT) in ethanol and processed for immunofluorescence microscopy. Control cells were treated with ethanol vehicle alone (-DHT). In Myr-CβDG-GFP expressing cells the GFP fluorescence (green in merge) can be seen at both the plasma membrane and in the nuclei of cells, whereas in the Myr-CβDGΔNLS-GFP expressing cells is at the plasma membrane or in a bright area outside the nucleus presumed to be the Golgi apparatus. In cells treated with DHT, the androgen receptor (red in merge) translocates robustly to the nucelus in both Myr-CβDG-GFP and Myr-CβDGΔNLS-GFP expresing cells, whereas in untreated cells the androgen receptor is

nuclear and cytoplasmic. Merged images are counterstained with DAPI (blue) to highlight the nucleus.
